# Application of Telepathology for Rapid On-Site Evaluation of Touch Imprint Cytology in CT-Guided Percutaneous Transthoracic Core Needle Biopsy of Pulmonary Nodules: The Experience of Our Multidisciplinary Thoracic Tumor Board

**DOI:** 10.3390/cancers17111738

**Published:** 2025-05-22

**Authors:** Stefano Lucà, Riccardo Monti, Carminia Maria Della Corte, Antonia Cantisani, Immacolata Cozzolino, Eduardo Clery, Martina Amato, Laura Marone, Francesca Capasso, Gaetano Di Guida, Beatrice Leonardi, Floriana Morgillo, Alfonso Fiorelli, Renato Franco, Marco Montella, Giovanni Vicidomini

**Affiliations:** 1 Department of Mental and Physical Health and Preventive Medicine, Pathology Unit, University of Campania “Luigi Vanvitelli”, 80131 Naples, Italy; 2 Department of Precision Medicine, Radiology Unit, University of Campania “Luigi Vanvitelli”, 80138 Naples, Italy; 3 Department of Precision Medicine, Medical Oncology, University of Campania “Luigi Vanvitelli”, 80131 Naples, Italy; 4 Department of Translational Medicine, Thoracic Surgery Unit, University of Campania “Luigi Vanvitelli”, 80131 Naples, Italygiovanni.vicidomini@unicampania.it (G.V.)

**Keywords:** digital pathology, telepathology, telecytology, Rapid On-Site Evaluation, touch imprint cytology, percutaneous transthoracic core needle biopsy, pulmonary nodules, non-small cell lung cancer

## Abstract

Background: Lung cancer remains the leading cause of cancer-related mortality, often diagnosed at advanced stages, where minimally invasive tissue sampling is essential for diagnosis and molecular profiling. Rapid On-Site Evaluation (ROSE) enhances the diagnostic yield of small biopsies, but is frequently limited by a shortage of pathologists and logistical constraints. Telepathology offers a potential solution by enabling remote real-time assessment. This study evaluates the feasibility, diagnostic accuracy, and efficiency of telecytology-assisted ROSE (TC-ROSE) using touch imprint cytology (TIC) during CT-guided transthoracic core needle biopsy (CNB) of pulmonary nodules. Methods: 50 patients underwent CNB. TIC samples were assessed and evaluated on-site or remotely via a fully remote-controlled microscope system (OCUS^®^). TIC slide preparation was performed by pathologists (30 cases), radiologists (10), and trained assistants (10). The study analyzed diagnostic concordance between remote and on-site assessments, time efficiency, and the feasibility of involving non-pathologists in TIC preparation. Results: Diagnostic samples were obtained in 86% of TIC samples, with full concordance (100%) between TC-ROSE and traditional ROSE. The slides required approximately 140 s for scanning, and the overall evaluation time was around 3 min per case. Overall, 100% of TICs were adequately assessed by both pathologists and non-pathologists. No increased number of complications was recorded among patients with TCROSE, compared to those ROSE evaluated. The remote setup allowed pathologists to maintain routine workflows, improving time efficiency. Conclusions: The findings confirm that telecytology is a viable, accurate, and efficient approach to ROSE, offering a practical solution for overcoming workforce and logistical barriers, particularly in settings with limited pathology resources.

## Simple Summary

Lung cancer requires effective tissue sampling. Rapid On-Site Evaluation (ROSE) enhances biopsy quality but is limited by a lack of pathologists and logistic barriers. This study tested telepathology-assisted Rapid On-Site Evaluation (ROSE) using touch imprint cytology (TIC) during CT-guided lung biopsies in 50 patients. TIC samples were assessed on-site or remotely via a remote-controlled microscope. We analyzed the population with both traditional ROSE and tele-ROSE, with full concordance between on-site and remote assessments. Telecytology overcame logistical challenges and optimized pathologists’ time. Though scanning time was a drawback, it did not impact procedural success. The findings support integrating telecytology-based ROSE into routine practice.

## 1. Introduction

Lung cancer (LC) is the most common malignancy and the leading cause of cancer-related mortality worldwide. Among its various histological subtypes, non-mucinous lung adenocarcinoma (NM-LUAD) is the most prevalent [1]. Most of the LCs are identified at advanced stages, making them ineligible for surgical intervention. Consequently, small tissue specimens (STSs) collected through minimally invasive techniques, including percutaneous core needle biopsy (CNB), transbronchial biopsy (TBB), and cell block (CB), are frequently the only samples available for diagnostic and therapeutic purposes [2].

CT-guided percutaneous transthoracic CNB of pulmonary nodules (PNs) is a well-established, standardized, and safe procedure for obtaining tissue to establish a diagnosis, even for small and “hard-to-reach” lesions, with a high diagnostic yield and great accuracy [3,4].

During percutaneous CNBs, the pathologist’s role is often required as support to the radiologist, so the idea of a Rapid On-Site Evaluation (ROSE) was developed [5]. ROSE is a technique that allows assessment of the adequacy of STSs and may be performed during a biopsy procedure from any anatomic site and, by employing a rapid staining method, a cytological slide can be prepared within minutes, allowing for immediate evaluation under a light microscope [6].

The use of touch imprint cytology (TIC) is strongly advised to be used for histological samples for on-site evaluation. A TIC preparation for ROSE entails that a histologic sample obtained using forceps, Tru-Cut biopsy, or cryobiopsy is placed on a microscope slide and is gently rolled over the slide. This releases cells to the slide and allows for subsequent rapid on-site examination [7,8,9].

An experienced cytopathologist is crucial for accurately evaluating collected material, formulating a preliminary diagnosis, and deciding on additional tests or further biopsies. Inadequate handling or equivocal on-site diagnoses can lead to premature termination of procedures and an increased need for further invasive procedures, negatively impacting patient care [10].

The shortage of pathologists, which is already responsible for inconvenient delays for diagnosis and treatment, joins the disadvantages of ROSE. We principally lack pathologists because of the low number of Medical Course Students (MCSs), which consider pathology as a possible future career, and the dropout of Post-Graduate Medical Schools (PGMS) [11]. In Italy, the current number of practicing pathologists is estimated to be approximately 2000, with a significant proportion nearing retirement age, which raises concerns about the sustainability of the workforce in the coming years [11]. Particularly, the ratio of pathologists to inhabitants is almost 1:30,000 [12].

Additionally, pathology departments are often centralized in a single location, leading to difficulties for professionals in the field. The wide distance from other departments where the pathologist is required (such as the ROSE in the Radiology Department) is another disadvantage for pathologists’ work. It can result in a complicated and time-consuming job, slowing down the quality and celerity of diagnoses [13]. To overcome these challenges, multiple AI-assisted telepathology platforms have been recently developed to support the process of ROSE. Yan et al. [14] developed an AI system based on a deep convolutional neural network to identify malignant cells in cytological slides during ROSE of flexible bronchoscopy. The system demonstrated a sensitivity greater than 90% for the detection of neoplastic cells and a high level of diagnostic agreement with expert cytopathologists, suggesting that this AI system can effectively support rapid sample evaluation during bronchoscopy. Other AI models, developed and validated, have demonstrated promising potential in replacing on-site cytopathological evaluation, thereby addressing the shortage of pathologists in numerous healthcare institutions [15,16,17]. However, the majority of these models do not specifically target pulmonary disease, and their clinical performance remains to be thoroughly validated within real-world practice settings. Otherwise, the first encouraging results regarding the application of telepathology to ROSE of pulmonary lesions came from the study by Naso et al. [18]. The authors evaluated the feasibility and diagnostic concordance of a robotic telecytology system (rmtConnect Microscope) for remote evaluation of bronchoscopic cytology specimens, including EBUS-FNA and transbronchial biopsy samples. The robotic system allowed pathologists to remotely assess specimen adequacy and provide diagnoses with a high degree of concordance compared to both non-robotic telecytology and traditional glass slide evaluations. Diagnostic agreement was strong, with a Cohen’s kappa of 0.84 between robotic and non-robotic platforms, and a median diagnostic time of just 85 s. Collaboration with radiologists and laboratory technicians can enhance the quality of healthcare delivery, especially in contexts where the shortage of pathologists may negatively impact diagnostic services. Indeed, Italy has a relatively high number of radiologists compared to other countries, with approximately 14,000 professionals, although the distribution is uneven, and certain regions experience deficits [19].

In addition, trained laboratory assistants and biomedical laboratory technicians play a vital role in diagnostic workflows; there are approximately 25,000 such professionals in Italy, although only a subset is specifically involved in pathology-related activities, progressively acquiring greater competence within the Pathology Unit in order to address the increasingly frequent shortage of pathologists [20]

## 2. Materials and Methods

### 2.1. Selection of Cases

This prospective study was conducted in collaboration with the Radiology Unit of “Università degli Studi della Campania Luigi Vanvitelli” Hospital in Naples, Italy, from September 2024 to January 2025.

We included 50 consecutive patients with a solitary lung nodule suspected of LC and eligible for a CT-guided lung CNB biopsy.

The study involved oncology specialists for patient recruitment, radiologist experts in pleuro-pulmonary pathology for biopsy procedures, thoracic surgery specialists to manage any complications, pathologist experts in cytology and pleuro-pulmonary pathology, and assistants in training in pathological anatomy. In addition, pathologist experts in ROSE received training on the use of TC-ROSE. Written informed consent was obtained for the 50 patients.

The study was preceded by an initial testing phase of the Ocus^®^20 device in the Pathological Anatomy laboratory and in the Interventional Radiology Service (Grundium Oy Saavutustenkatu 3 33720 Tampere Finland). Supervision and support were provided by the Information Technology (IT) Service, the Office for the Digital Transition of the University of Campania “L. Vanvitelli”, and the Specialists of the device in use.

All digital data were transmitted within the University/Hospital network, in accordance with the General Data Protection Regulation (GDPR) regulations of the European Union (EU).

**Figure 1:**
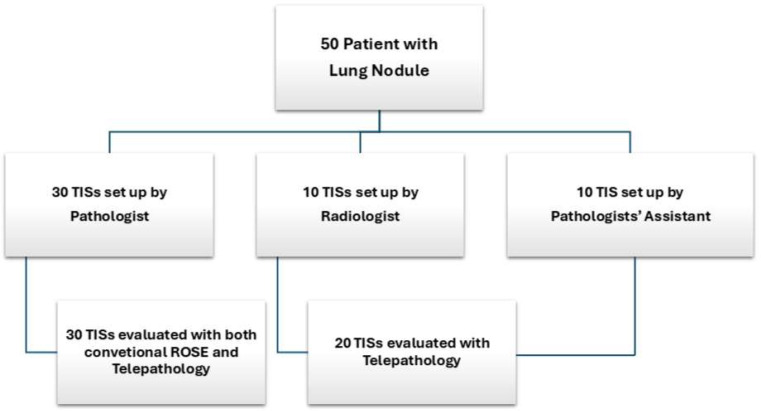
Schematic diagram about the method of preparation and evaluation of TISs. TIC and staining were performed by a pathologist, radiologist, and pathologist’s assistant and evaluated with both conventional TIC-ROSE or telepathology or only with telepathology.

The study was divided into 3 phases (Figure 1):

Phase 1 (30 patients): traditional ROSE and TC-ROSE, with TIC setup by the pathology anatomy staff;

Phase 2 (10 patients): TC-ROSE, with TIC setup by the interventional radiology staff and supervision by a pathologist;

Phase 3 (10 patients): TC-ROSE, with TIC setup by assistants in training and supervision by a pathologist.

### 2.2. Overview of the Biopsy Procedure

A percutaneous transthoracic core needle biopsy (CNB) was performed under computed tomography (CT) guidance by a radiologist with support from a pathologist. The procedure utilized a 64-slice CT scanner (Siemens SOMATOM go.Top) to acquire thoracic images from the lung apices to the diaphragm, enabling accurate localization of the target lesion. Needle placement was guided by CT imaging, and patient positioning was adjusted as needed to optimize access, minimizing interference with adjacent structures such as large vessels, bronchi, or fissures. The entry site was determined using external CT laser markers and skin-based anatomical references. Patients were instructed to maintain the same position and suspend breathing at key procedural moments.

Local anesthetic was administered subcutaneously, avoiding pleural penetration. A coaxial biopsy technique was employed, using an 18-gauge semiautomatic needle (Speedybell, Biopsybell). The coaxial cannula was advanced to the lesion’s edge, and the biopsy needle, equipped with a spring-activated cutting system, was deployed to extract tissue samples in 10 or 20 mm segments. Each pleural puncture allowed for up to four samplings.

Following the biopsy, a CT scan was performed to detect immediate complications, such as pneumothorax or hemorrhage. Additionally, post-procedure chest radiographs (PA view, upright inspiration) were obtained at both 2 and 24 h to monitor for delayed complications prior to patient discharge.

### 2.3. Scanning System

According to the OCUS^®^ Datasheet, the characteristics of the device are as follows:

Resolution microscope 0.5 µm/pix, Depth of focus 5 µ, Focusing fully ekectronic, Image sensor 12 Mpix, connectivity 1 GigE, 802.11 ac WLAN, image formats TIFF and SVS, W × D × H 18 × 18 × 19 cm, weight 2.5 kg

### 2.4. ROSE and TCROSE Procedure

**Figure 2:**
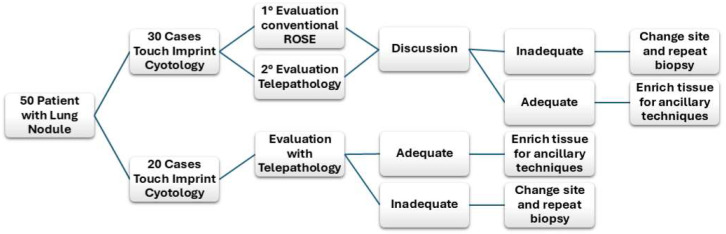
More detailed and schematic flow chart about the TIS evaluation method. The adequacy of 30 cases was evaluated by two pathologists (on-site and remotely), while 20 cases were assessed exclusively remotely by one pathologist. Based on the pathological interpretation, we decided whether to eventually enrich the biopsy material or change the biopsy site.

**Figure 3:**
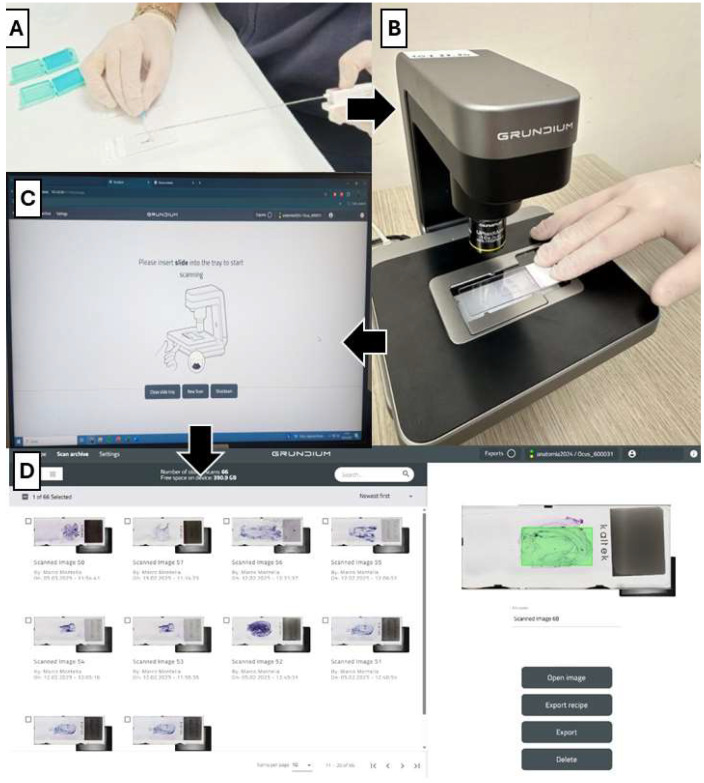
Telepathology TIC-ROSE: different steps of the process. (**A**) TIS preparation; (**B**) Image transmission; (**C**) Selection of scanning area; (**D**) Scanning complete.

The first biopsy obtained from each patient was used for ROSE evaluation. The tissue core was imprinted over the glass for TIC, and the smear was air-dried and Diff Quik-stained for cytologic evaluation. TIC and staining were performed in the first 30 cases (60%) by a pathologist, and the touch imprint slides (TISs) were evaluated by on-site and simultaneously scanned by a microscope slide scanner (OCUS^®^) for remote evaluation by a second pathologist. Regarding the subsequent 20 cases (40%), TISs were randomly setup by a radiologist (10/20, 50%) and by a pathologist’s assistant (10/20, 50%), and the evaluation was performed exclusively remotely (Figure 1). Immediately after the imprinting, the core biopsy was fixed in 4% formalin for further histologic processing and paraffin embedding. In detail, a second pathologist evaluated, “live viewing”, the most cellular area and scanned it for more accurate evaluation via remote control of the microscope. The size of the scanned area was measured, and the time required for scanning and morphological assessment of adequacy was recorded. The adequacy of the TIC was assessed according to the following criteria: presence of >200 cells, concordance with the clinical/instrumental suspicion, appropriate morphological evaluability, and absence of excessive contaminating elements (necrosis, blood, etc.) [21]. In total, 20 cases were evaluated by a single pathologist remotely, while in 30 cases, the evaluation was performed by two pathologists, one of which on-site and the other remotely, and they compared their findings and discussed any discrepancies by phone. Finally, if the case was deemed adequate for a diagnosis, additional samples were obtained from the mass to enrich the biopsy material and ensure a sufficient tissue amount required for ancillary techniques, such as immunohistochemistry or molecular biology. On the other hand, if the case was qualified as inadequate for a diagnosis, sampling was repeated, eventually by changing the biopsy site, until adequacy was achieved (Figure 2). Figure 3 summarizes the sequence of events from biopsy sampling to evaluation by CT-ROSE.

### 2.5. Statistical Analysis

Data were collected by comparing the TC-ROSE assessment of sample adequacy—defined in terms of qualitative adequacy (evaluable cellular morphology), quantitative adequacy (>50 diagnostic cells per smear), and sufficient sample volume—with the final diagnostic report (categorized as adequate or inadequate). The criteria employed for case classification are detailed in Table 1.

Sensitivity was calculated as TP/(TP + FN) and specificity as TN/(TN + FP).

Precision, or positive predictive accuracy, was defined as the proportion of cases classified as adequate by TC-ROSE that were confirmed as adequate in the final diagnosis, using the formula TP/(TP + FP).

Overall accuracy was calculated as the proportion of correctly classified cases—both adequate and inadequate—by TC-ROSE and final histology, using the formula (TP + TN)/(TP + TN + FP + FN).

The positive predictive value (PPV) was calculated as TP/(TP + FP) and the negative predictive value (NPV) as TN/(TN + FN).

## 3. Results

### 3.1. Clinico-Radiological Features

Clinico-radiological features of our series are summarized in Table 2. The 50 cases include 18 female patients (36%) and 32 male patients (64%), ranging from 52 to 80 years with an average age of 69 years.

In 28 out of 50 cases (56%), the lesion involved the right lung, while 22 cases (44%) were located in the left lung. In detail, 15 out of 28 (53.5%) were located at the right upper lobe (RUL), 12 out of 28 (42,8%) at the right lower lobe (RLL), and 1 case (1/28, 3.7%) at the right middle lobe (RML). The lesions identified in the left lung were instead distributed as follows: 12 out of 22 (54.5%) at the left lower lobe (LLL), 9 out of 22 (41%) at the left upper lobe (LUL), and 1 case (4.5%) at the lingula.

The TC major axis of the pulmonary nodules ranged from 10 to 75 mm, with an average size of 42.2 mm.

With respect to the complication rate, mild perilesional hemorrhage was the most commonly observed event (15 out of 50, 30%), whereas pneumothorax occurred in 7 cases (7/50, 14%). The overall complication rates were consistent with those reported in the current literature [22,23,24,25,26,27] and the procedure was not interrupted due to complications in any case (Table 2).

We did not experience a difference in terms of complications among patients who underwent traditional ROSE vs. TC-ROSE

### 3.2. Pathological Features

Pathological features of our series are summarized in Table 3. All lung lesions underwent CT-guided percutaneous transthoracic CNB, and in no instance was the procedure interrupted due to complications. The biopsy fragments range from 4 to 76 mm in size, with an average size of 26 mm.

**Figure 4:**
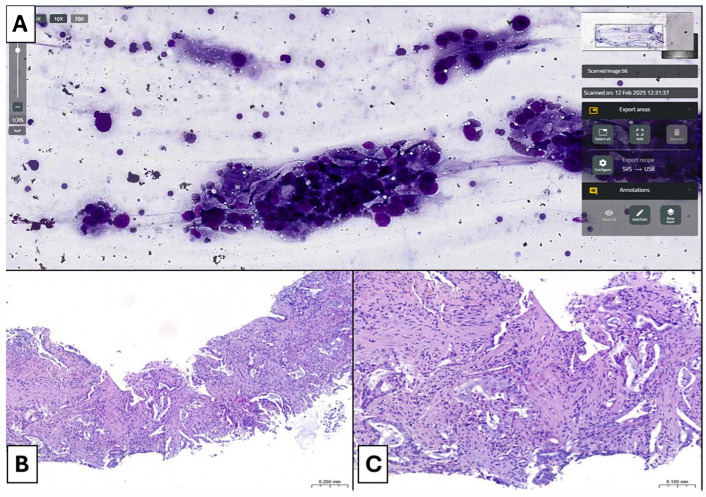
Scanned TIS and corresponding lung core biopsy with a diagnosis of NSCLC-type adenocarcinoma. (**A**) Scanned TIS shows clusters of or single large epithelioid cells characterized by significant cytological atypia and pleomorphism. Neoplastic elements show an increased nucleus-to-cytoplasm ratio, with large and irregular nuclei. (Diff-Quick staining, original magnification: 10×); (**B**,**C**) Lung core biopsy shows tissue invasion by adenocarcinoma. Acinar and fused gland patterns are observed with neoplastic cells characterized by a large and mildly eosinophilic or amphophilic cytoplasm and huge and irregular nuclei with hyperchromasia or finely dispersed chromatin. A mucoid material is appreciable. In this case, both morphological and immunohistochemical features allowed the diagnosis of NSCLC of adenocarcinoma type ((**B**) H&E staining, original magnification: 4×; (**C**) H&E staining, original magnification: 10×).

**Figure 5:**
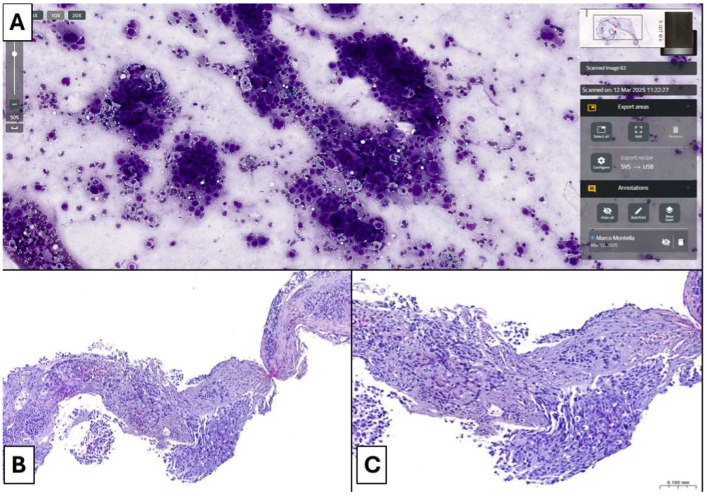
Scanned TIS and corresponding lung core biopsy with a diagnosis of NSCLC-NOS. (**A**) Scanned TIS shows clusters of large epithelioid cells characterized by evident anisonucleosis and significant cytological atypia. Abundant cytoplasms are observed but a significantly increased nucleus-to-cytoplasm ratio is appreciated, with huge and irregular nuclei. Rare anaplastic cells are appreciable. The slide was considered adequate for diagnosis (Diff-Quick staining, original magnification: 10×); (**B**,**C**) Lung core biopsy shows tissue invasion by nests of poorly differentiated epithelial neoplasm. Tumor cells are large and show significant pleomorphism, a cytological atypia. Evident amphophilic cytoplasm and irregular, huge, and hyperchromatic nuclei are visible. In this case, no morphological, histochemical, or immunohistochemical features allowed for a precise definition of the histological type (only CK7 expression by tumor cells), so the final diagnosis was NSCLC-NOS ((**B**) H&E staining, original magnification: 4×; (**C**) H&E staining, original magnification: 10×).

The surface area of the TISs to be scanned measured from 3 × 1.5 mm to 20 × 18 mm, with an average size of 11 × 9.75 mm. This average size corresponded to an average TSI scanning time of 140 s (Table 3). Obviously, the scanning time was directly proportional to the surface area of the slide to be scanned, exceeding 200 s for slides with a large scanning area. Most TISs (43/50, 86%) were considered adequate for a subsequent histopathological diagnosis (Figure 4 and Figure 5), and only 7 out of 50 cases (14%) were interpreted as adequate for a histopathological diagnosis but not representative of the clinically suspected disease. No TISs were deemed inadequate for a histopathological diagnosis. Regarding the 30 cases simultaneously evaluated by two pathologists (one on-site and one remotely), we observed full concordance (100%) between their assessments for all patients (Table 4).

We performed a histopathological diagnosis on the respective CNBs of all the “adequate” TISs (43/50, 86%) (Figure 4 and Figure 5) (Table 5). In detail, the histopathological diagnosis included 37 (37/43, 86.1%) Non-Small Cell Lung Cancer (NSCLC) of which 27 (27/37, 73%) were lung adenocarcinomas (LUADs), 5 of which (5/27, 18.5%) with mucinous features, 9 (9/37, 24.3%) were squamous cell carcinomas of the lung (LUSCs) and 1 (1/37, 2.7%) was a non-small cell carcinoma NOS (NSCLC-NOS); 1 (1/43, 2.3%) neuroendocrine tumor (NET-NOS); 1 (1/43, 2.3%) solitary fibrous tumor of the pleura (SFT); and 4 (4/43, 9.3%) lung metastases (3 from colorectal adenocarcinoma and 1 from bladder urothelial carcinoma). Of the 7 cases (7/50, 14%) deemed adequate but not representative of the clinically suspected disease on ROSE, 5 (5/7,71.4%) showed features of pulmonary inflammatory process with reactive pneumocyte hyperplasia and, sometimes, some peculiar histopathological features, while 2 cases (2/7, 28.6%) were diagnosed as LC. Specifically, histopathological diagnosis was LUAD in 1 case (1/7, 14.3%) and NSCLC-NOS in the other case (1/7, 14.3%) (Table 5).

### 3.3. Statistical Analysis

Regarding the TC-ROSE analysis, 48 True Positive cases were observed, no (n.0) True Negative cases, no (n.0) False Positive cases, and 2 False Negative cases, which therefore highlighted the following:

Sensitivity 96%;

Specificity 100%;

Precision 100%;

Accuracy 96%;

Positive predictive value 100%;

Negative predictive value 0%.

## 4. Discussion

Minimally invasive tissue acquisition using percutaneous CNB is mandatory for the diagnostic definition of a pulmonary lesion and for leading therapeutic decisions in the era of precision medicine. In this context, the opportunity to work with lung biopsy samples provides many benefits [28,29,30,31,32,33]. The quantity and quality (structural integrity) of tissue obtained by CNB allow for an accurate histopathological diagnosis, both for benign and malignant pulmonary neoplasms, and to suspect a non-neoplastic pulmonary disease. Furthermore, adequate tissue sampling is crucial in determining the molecular status of the neoplasm, in identifying driver and targetable mutations, and in assessing the expression levels of PD-L1 [33,34].

In this context, the use of an introducer coaxial needle is another significant advantage of CNB, as it works as a guide and allows for the acquisition of multiple tissue samples from the same appropriate biopsy site [35]. Collaboration between the radiologist and pathologist is essential to confirm the appropriate biopsy site, and the pathologist plays a crucial role in assessing the adequacy of the biopsy sample through on-site evaluation [36]. The enhancement of sample adequacy and diagnostic precision, reflected in reduced rates of inconclusive and ambiguous diagnoses, along with an increase in diagnostic yield and sensitivity, is recognized as a key benefit of the ROSE [10]. Strictly numerical data regarding the impact of ROSE are obviously very variable in the literature; however, a recent Chinese study conducted in pulmonary pathology has highlighted that the positive rate of biopsy in the ROSE group is 84% and the controls is 74%, with an increase of 10% of successfully performed biopsy procedures [37]. Nevertheless, this technique presents certain challenges. Specifically, its implementation is dependent on institutional resources and logistical factors, as it necessitates investment in time, financial resources, and specialized personnel [10,38].

At our institution, CT-guided percutaneous trans-thoracic CNBs and corresponding ROSE are performed weekly, typically involving the presence of a well-trained pathologist on-site during sampling. The pathologist is responsible for macroscopically evaluating the biopsy specimens, preparing touch imprints on a glass slide, rapidly staining the slide using Diff Quick staining, and microscopically assessing the adequacy of the CNB without requiring a definitive diagnosis. Concurrently, the biopsy is immediately placed between two sponges in a bio-cassette, which is then immersed in a container filled with 10% neutral buffered formalin to prevent rapid degradation of tissue proteins and nucleic acids, before being transported to the pathology laboratory at room temperature. Obviously, although we consider ROSE an unquestionably advantageous technique, we also experience its inherent drawbacks. Principally, the need for experienced on-site professionals and the ratio between time consumed/n. of diagnosis, especially if there are logistical issues. The time dedicated to the analysis is taken away from the “conventional” routine diagnostic activities, a situation further compounded by the significant distance between our Pathology Department and the Radiology Unit, which unfortunately are not located within the same facility. The lack of extra time for the procedure, combined with the shortage of pathologists, may negatively impact the timeliness of routine diagnostic activities. One potential approach to optimizing pathologists’ time and addressing obstacles to widespread integration of on-site evaluation involves leveraging trained assistants or pathology residents for initial setup, as well as incorporating telepathology. Telecytology, a subset of telepathology, facilitates the remote assessment of cytological images for purposes such as primary diagnosis, teleconsultations, and ROSE [28,29,30,31,32,39].

Telecytology platforms operate via either static or dynamic image transmission [40,41].

“Static systems” involve capturing and forwarding pre-selected images (such as photographs or whole-slide images), whereas “dynamic systems”, including video streaming and robotic microscopy, provide real-time visualization of cytological material.

Despite the differences in transmission methods, all telecytology systems share four essential components:

1. Imaging devices at the site, including a camera, supporting hardware, and software, to create digital representations of samples.
2. A dedicated software platform that enables remote transmission of the captured images;
3. A network communication system, such as an internet connection or Wi-Fi, to facilitate data transfer;
4. A remote viewing station, consisting of a monitor, necessary hardware, and software, for analyzing the received images

These components establish a comprehensive pixel pathway from image capture to remote display, necessitating validation studies that assess the entire workflow holistically rather than evaluating each element independently.

Among the most common and straightforward methods for image acquisition is the use of a digital camera affixed to a microscope [42]. While cost-effective and relatively simple, this method requires an on-site operator with cytopathology expertise to manipulate the microscope, locate relevant regions, and capture representative images in static systems. In contrast, robotic microscopes eliminate the need for an on-site cytopathology-trained operator by enabling remote control of microscope functions, including objective selection, slide navigation, and focusing [29].

Each telecytology system has distinct advantages and limitations in terms of availability, usability, focusing capabilities, speed, costs, on-site personnel requirements, and institutional approval. Although robotic microscopy reduces reliance on on-site specialists, it demands specialized equipment, incurs higher costs, and may be affected by bandwidth constraints due to large image file sizes.

Additionally, image analysis using robotic microscopes is typically slower, as it follows a process similar to whole-slide imaging, which has been shown to be less efficient than traditional glass slide review [29]. Consequently, it is essential to recognize that robotic microscope-based telecytology may be slower than dynamic telecytology assisted by an on-site trained operator. Telecytology enables the timely assessment of cytological specimens, maintaining diagnostic accuracy while reducing procedure times. This approach could facilitate efficient communication between on-site clinicians and remote pathologists, enhancing workflow and patient care in interventional procedures [43].

To the best of our knowledge, only one study has applied dynamic telecytology to imprint cytology samples from CT-guided lung biopsies. In this study, the radiologist operating the microscope achieved a diagnostic accuracy of 100%, as cytologists arrived at the correct diagnosis for all samples. However, when the radiologist’s technician operated the microscope, two diagnoses made by Cytologist 1 differed from the gold standard, resulting in an accuracy of 95.56% for the technician. In our experience, the accuracy was 96%.

In our routine practice, we have managed to avoid this pitfall since the device at our disposal is fully remotely controlled. This has allowed us to perform both a scan of the requested area and a live view when necessary. However, it should be noted that the live view is not as fast as a traditional microscope, and the speed of image transmission is directly proportional to the available internet bandwidth.

In our experience, we believe that the application of telepathology for the evaluation of sample adequacy is very promising. Telecytology has shown diagnostic accuracy comparable to on-site evaluation (Table 4). Even when used alone, the diagnostic suspicion provided by scanned TSIs has always been confirmed on biopsy samples. Obviously, this approach is not without its challenges. A potentially critical issue is the scanning time. The scanning time is proportional to the area of the surface to be scanned. In our series, the scanning area ranged from a minimum of 3 × 1.5 mm to a maximum of 20 × 18 mm. We observed that the scanning area providing the optimal compromise between assessable cellularity and scanning time (approximately 120–130 s) was approximately 10 × 10 mm.

However, it has not been demonstrated that waiting time negatively affects the biopsy procedure, the complication rate, or the quality of the collected sample, even in the most critical cases, characterized by procedural delays with scanning times exceeding 200 s. In this regard, only 5 cases (5/50, 10%) had a scanning time exceeding 200 s; all these cases (5/5, 100%) were deemed adequate and subsequently diagnostic. Mild perilesional hemorrhagic suffusion was observed as a complication in only two of them (2/5, 40%). This analysis leads us to two conclusions: first, imprint cytology, compared to conventional cytology, has the advantage of allowing control over the surface area of the biopsy fragment’s imprint, ensuring it remains within the recommended limits. Second, the activity of a pathologist’s assistant, pathology resident, or radiologist adequately trained in TIS preparation has been effective in minimizing unnecessary and time-consuming procedures.

Our average total time to assess the adequacy of the biopsy specimen was approximately 3 min per case. Usually, we perform three to four CT-guided percutaneous transthoracic CNBs per session; thus, the total time required for the pathologist’s activity is a maximum of approximately 12 min, rather than hours of on-site evaluation. Therefore, the proposed approach is expected to remain efficient even in high-volume centers, due to the relatively short time required for TIS evaluation, approximately three minutes per biopsy. This brief evaluation time ensures that high workloads and workflows do not negatively impact the effectiveness of the approach. Any potential bottleneck in remote pathologist availability during peak hours can be managed through optimized work scheduling.

Going into the details of savings, the College of American Pathologists (CAPs) estimates that the average hourly wage for a pathologist in the United States is USD 151, with a range of USD 108 to USD 205 per hour, depending on experience and location. In Italy, the average hourly wage for a pathologist is around EUR 47, with a range of EUR 22 to EUR 74 per hour, considering a 40-h work week [44].

In the United States, wages are generally higher, with significant variations between states, while in Europe, wages vary between countries, with Germany offering the highest wages.

Another survey, also published in Pathologica, highlighted a shortage of pathologists in Italy, with each professional responsible for around 3000 diagnoses per year. If there are 250 working days in a year, with approximately 2000 h/year, on average, it is estimated that a pathologist performs 1.5 diagnoses/h.

Considering that the average time for a biopsy procedure requires approximately 40–50 min per patient, it follows that for a biopsy session, approximately 3 h of work are necessary, plus the travel necessary to reach the structure of the interventional procedures, which in our case requires approximately 1 h there and 1 h back.

A pathologist engaged in a biopsy shift, in our experience, would spend approximately EUR 295, with a diagnostic delay of approximately seven to eight diagnoses per work shift.

Moreover, Dahlberg et al. [45] have demonstrated that Rapid On-Site Evaluation (ROSE) during navigational bronchoscopy for pulmonary nodules is a cost-effective approach and advocated for its broader adoption in clinical practice. However, widespread implementation is hindered by financial disincentives—namely, the under-reimbursement by third-party payers, who typically fall short by USD 40 to USD 50 per billing code. This shortfall shifts the financial burden onto hospitals, complicating their resource allocation strategies. Adjustments to reimbursement policies could enhance the viability of ROSE programs by offering institutions stronger financial motivation to adopt them. Notably, ROSE would remain a cost-effective option unless its cost per procedure exceeded USD 988 [45].

This represents a significant advantage as it eliminates downtime and allows for the maximization of the pathologist’s working time. Another significant result achieved was that following appropriate training of the radiologist or pathologist’s assistant; no significant differences were observed in the quality of TIS preparation when compared to that performed by the pathologist.

This result is by no means intended to undermine the professionalism of any healthcare professional or to overburden one role at the expense of another. Rather, it aims to open the mind to alternative solutions in cases where a healthcare facility may lack enough pathologists or pathologists’ assistants. As always, the cornerstone of well-executed work is communication among specialists, allowing them to share the know-how of a procedure to better support their colleagues and ensure optimal procedural efficiency.

## 5. Conclusions

TIC-ROSE of CT-guided percutaneous transthoracic CNB allows for the enhancement of diagnostic accuracy and precision, resulting in reduced rates of unsatisfactory and suspicious diagnoses and ensuring an increased diagnostic yield. ROSE is one of the various applications of telepathology. Telepathology TIC-ROSE can improve pathology practice by increasing productivity and reducing costs in regions with limited access to pathologists. Additionally, proper training of laboratory technicians and/or radiology staff ensures the correct preparation and scanning of slides, enabling remote evaluation by the pathologist. This optimizes the adequacy assessment during the sampling procedure, maximizing the number of diagnostic biopsies and ensuring adequate tissue sampling, which is essential for determining the molecular status of the neoplasm and identifying driver and targetable mutations in the era of precision medicine. The proposed approach could equally be applied to the adequacy assessment of other biopsy types, such as TransBronchial Biopsies (TBBs), Ultrasound-guided lung or mediastinal lymph node Cryobiopsies, or EndoBronchial UltraSound-guided TransBronchial Needle Aspiration (EBUS-TBNA). Furthermore, this model can be transferred to other anatomical sites, specifically in the case of core biopsies performed for breast or kidney disease, or EUS-Fine-Needle Aspiration Biopsy (EUS-FNAB) of pancreatic masses. In this case, however, some adjustments might be necessary, considering that some diagnostic procedures require different sampling techniques. An example could be the different types of training required for TIS preparation based on the different types of small samples collected. The type of medical center could also obviously influence the application of the model; in high-capacity centers, for example, where technological resources and expertise are more abundant, remote evaluation could be easily integrated, while in rural clinics, the limited availability of advanced equipment and specialized personnel could pose a barrier. So, all that glitters is not gold. Our study has some limitations; firstly, the small number of cases. By increasing the number of patients evaluated, a greater discrepancy between TIC-ROSE and conventional ROSE might be observed. Moreover, remote evaluation relies on a well-functioning and efficient network connection, and connection issues can negatively affect the procedure, potentially making the evaluation impossible. The definition of shared recommendations, also about an appropriate training of radiologists, pathology residents, or pathologist’s assistants in the preparation of TIC-ROSE, could certainly improve the implementation of the telepathology TIC-ROSE in the clinical routine. However, even if there are currently no specific validation guidelines to ensure safe practice, our results appear promising, suggesting that this approach could be successfully integrated into clinical practice.

## Figures and Tables

**Table 1:** Category for calculating statistical analysis.

Category	Adequacy
True Positive	TC-ROSE evaluation matched the final report on bioptic sample
True Negative	TC-ROSE evaluation of inadequacy matched the final report on bioptic sample
False Positive	TC-ROSE opinion was deemed adequate, but the final report on bioptic sample was inadequate or insufficient for diagnosis
False Negative	TC-ROSE opinion was inadequate, but the final report on bioptic sample was adequate and diagnostic

**Table 2:** Clinico-radiological features of the series.

Clinico-Radiological Features	N° Cases (%)
Sex	
Male	32/50 (64%)
Female	18/50 (36%)
Years	
≤60	9/50 (18%)
>60	41/50 (82%)
Mean	69
Median	72
CT Diameter (mm)	
Median	40
Mean	42.2
Side	
Right Lung	28/50 (56%)
Left Lung	22/50 (44%)
Site of the Lesion	
Right upper lobe (RUL)	15/28 (53.5%)
Right lower lobe (RLL)	12/28 (42.8%)
Right middle lobe (RML)	1/28 (3.7%)
Left upper lobe (LUL)	9/50 (41%)
Left lower lobe (LLL)	12/22 (54.5%)
Lingula	1/22 (4.5%)
Comorbidities	
COPD	15/50 (30%)
Previous Neoplasms (other district)	6/50 (12%)
Previous Neoplasms (thoracic district)	0/50
Sleep Apnea	4/50 (8%)
Dyslipidemia	18/50 (36%)
Osteoporosis	5/50 (10%)
Anemia	6/50 (12%)
Arterial Hypertension	22/50 (44%)
GERD	8/50 (16%)
Cardiopathy	4/50 (8%)
Smoking habits	
Smoker	18/50 (36%)
Non-smoker	10/50 (20%)
Ex-smoker	11/50 (22%)
N/A	11/50 (22%)
Complications	
Pneumothorax	7/50 (14%)
Pulmonary Hemorrhage (mild perilesional hemorrhagic suffusion)	15/50 (30%)
Hemoptysis	0/50 (0)
Hemothorax	0/50 (0)
Air Embolism	0/50 (0)

**Table 3:** Pathological features of the series.

Pathological Features	N° of Cases (%)
ROSE	
Adequate for diagnosis	43/50 (86%)
Not adequate for diagnosis	0/50 (0)
Adequate for diagnosis but not representative of the clinical suspect	7/50 (14%)
Surface of TIS (mm)	
Mean surface	11 × 9.75 mm
Scanning time (s)	
Mean time	140 s
Diagnosis	
Neoplastic	45/50 (90%)
Not neoplastic	5/50 (10%)
Histopathological Diagnosis	
NSCLC, Adenocarcinoma	23/50 (46%)
NSCLC, Adenocarcinoma with mucinous features	5/50 (10%)
NSCLC, SCC	9/50 (18%)
NSCLC-NOS	2/50 (4%)
NET-NOS	1/50 (2%)
SFT	1/50 (2%)
Metastasis	4/50 (8%)
Pneumocyte hyperplasia, probably reactive, with inflammatory background and respiratory bronchiolitis features	3/50 (6%)
Pulmonary inflammatory disease with organizing pneumonia features	1/50 (2%)
Pulmonary inflammatory disease with necrotizing granulomatous pneumonia features	1/50 (2%)

**Table 4:** Level of agreement between the assessments of the two pathologists.

	Evaluation	
	Adequate (%)	Adequate But Not Representative (%)
On-Site	27 (90%)	3 (10%)
Telepathology	27 (90%)	3 (10%)

**Table 5:** Agreement between ROSE and histopathological diagnosis on CNB.

ROSE	Histopathological Diagnosis on CNB						
	LUAD (%)	LUSC (%)	NSCLC-NOS (%)	NET-NOS (%)	SFT (%)	Lung Metastases (%)	Inflammatory Process (%)
Adequate	27 (62.8%)	9 (21%)	1 (2.3%)	1 (2.3%)	1 (2.3%)	4 (9.3%)	0
Adequate but not Representative	1 (14.3%)	0	1 (14.3%)	0	0	0	5 (71.4%)
Not Adequate	0	0	0	0	0	0	0

## Data Availability

No new data were created or analyzed in this study. Data sharing is not applicable to this article.

## Abbreviations

CT	Computed Tomography
EU	European Union
EUS-FNAB	EUS-Fine-Needle Aspiration Biopsy
FN	False Negative
FP	False Positive
GDPR	General Data Protection Regulation
IT	Information Technology
LC	Lung Cancer
LUSC	Lung Squamous Cell Carcinoma
MCS	Medical Course Students
NET	NeuroEndocrine Tumor
NM-LUAD	Non-Mucinous Lung Adenocarcinoma
NOS	Non Otherwise Specified
NPV	Negative Predictive Value
NSCLC	Non Small Cell Lung Cancer
PGMS	Post-Graduate Medical Schools
PN	Pulmonary Nodules
PPV	Positive Predictive Value
ROSE	Rapid On-Site Evaluation
SFT	Solitary Fibrous Tumor
STS	Small Tissue Specimen
TBB	Trans Bronchial Biopsy
TC-TOSE	Tele-Cytology Rapid On-Site Evaluation
TIC	Touch Imprint Cytology
TIS	Touch Imprint Slide
TN	True Negative
TP	True Positive
TBB	TransBronchial Biopsy
EBUS-TBNA	UltraSound-guided TransBronchial Needle Aspiration
